# Psychological Versus Somatic Correlates of Adolescent Suicide Attempts: A 21-Year National Time-Series and Path-Analytic Study in South Korea (2005–2025)

**DOI:** 10.3390/healthcare14162643

**Published:** 2026-08-20

**Authors:** Hyeran Jung, Minsun Jung

**Affiliations:** Department of Pathology, Yonsei University College of Medicine, 50-1 Yonsei-ro, Seodaemun-gu, Seoul 03722, Republic of Korea; phdgrace@yuhs.ac

**Keywords:** adolescent suicide, perceived stress, depression, atopic dermatitis, obesity, secular trend, ecological study, Korea Youth Risk Behavior Survey, structural equation modeling, time-series analysis

## Abstract

**Background:** South Korea reports one of the highest adolescent suicide rates among OECD countries. Both psychological factors (perceived stress, depression) and somatic/behavioral factors (atopic disease, obesity, diet, physical activity) have been proposed as correlates of adolescent suicidality, but the extent to which their co-occurring long-term national trends reflect genuine, independent associations—rather than a shared secular (time) trend—has rarely been tested at the population level. **Methods:** We compiled national annual prevalence estimates (2005–2025) from the Korea Youth Risk Behavior Survey (KYRBS), disseminated via the Korean Statistical Information Service (KOSIS), for eleven indicators. We computed (1) raw Pearson correlations, (2) year-detrended partial correlations, (3) multiple and hierarchical regression, and (4) an exploratory observed-variable path model on the year-adjusted series. **Results:** In raw correlations, nearly all indicators were significantly associated with the suicide attempt rate (|r| = 0.30–0.92). After removing the shared time trend, only perceived stress (partial r = 0.82, *p* < 0.001) and depressive mood (partial r = 0.81, *p* < 0.001) remained strongly and independently associated with suicide attempts. Detrended residuals were approximately normal (Shapiro–Wilk *p* > 0.26), and the two psychological associations were confirmed by Spearman rank-based partial correlations and permutation tests (both *p* ≤ 0.0002). A joint regression (suicide ~ stress + depression, year-adjusted) explained 93.5% of variance (adjusted R^2^ = 0.923), and stress and depression added significant explanatory power beyond calendar year (ΔR^2^ = 0.229, *p* < 0.001). **Conclusions:** Among the indicators examined, perceived stress and depressive mood show a robust population-level association with adolescent suicide attempts that is not attributable to a shared secular trend, whereas most somatic and behavioral correlates do not. The path model is exploratory and descriptive only and does not test causal mechanisms. Findings are ecological (population-level) and cannot establish—or exclude—individual-level causation.

## 1. Introduction

Adolescence is a period of heightened vulnerability to mental health difficulties, and South Korea has consistently reported one of the highest youth suicide rates among Organisation for Economic Co-operation and Development (OECD) member countries [1]. Suicide-related behaviors among Korean adolescents have been extensively studied using the Korea Youth Risk Behavior Survey (KYRBS), with correlates including perceived stress [2], adverse life events [3], short sleep duration [4], and energy drink consumption [5]. The sections below summarize the evidence linking somatic conditions and psychological factors to adolescent suicidality, beginning with Korean studies and extending to Asian and international evidence, with emphasis on work published since 2020, before setting out the rationale for the present analysis.

### 1.1. Somatic Conditions and Adolescent Mental Health

A substantial body of the literature links somatic and behavioral conditions to adolescent mental health. Chronic somatic conditions can increase psychological burden through symptom load, stigma, sleep disruption, and impaired social functioning, and adolescents with such conditions report higher rates of depressive symptoms and suicidality than their peers. Because the national prevalence of many of these conditions has changed markedly in Korea over the past two decades, they are frequently examined as candidate population-level correlates of adolescent suicide.

### 1.2. Atopic Disease and Psychological Distress/Suicidality

Atopic dermatitis has been associated with depressive symptoms and suicidal behaviors in Korean adolescents at the individual level [6], a relationship supported by a recent systematic review and meta-analysis reporting elevated odds of stress, depression, anxiety, and suicidal ideation among patients with atopic dermatitis [7]. Psychological stress and atopic disease also appear to be bidirectionally related [8]. Allergic diseases more broadly—including allergic rhinitis and asthma—have been associated with anxiety and depressive symptoms among Korean youth [9].

### 1.3. Obesity, Body Image, and Depression/Suicide Risk

Body weight, weight perception, and obesity [10], as well as physical-activity patterns [11], have likewise been linked to depression and suicidality among adolescents. Adverse weight perception and body image distortion, in particular, have been associated with depressive symptoms independently of measured body mass in Korean youth [10].

### 1.4. Perceived Stress and Depressive Symptoms as Proximal Predictors

Perceived stress and depressive mood have been repeatedly identified as proximal psychological correlates of suicidal ideation and attempts [2,12]. In individual-level KYRBS analyses, stress and depressive symptoms are among the strongest and most consistent correlates of suicide attempts, and comparisons before and during the COVID-19 pandemic identified them as central to changes in adolescent suicidality [12].

### 1.5. Potential Pathways and the Present Study

The literature establishes models in which somatic conditions act partly through psychological distress—for example, atopic disease or obesity increasing perceived stress and depressive mood, which in turn raise suicide risk—prompting increasingly complex explanatory models, including structural equation models, of how multiple risk factors jointly shape adolescent mental health [13]. However, most national-level analyses report a single survey wave or a small number of waves, and few explicitly test whether co-occurring long-run trends in mechanistically distinct indicators are genuinely associated with suicide-related outcomes, or whether they merely share a common secular trajectory across calendar time. This distinction is consequential. When two national indicators drift in parallel over two decades for unrelated reasons—changing survey methodology, generational shifts in reporting, or independent public-health interventions—a raw correlation between them is not evidence of a substantive relationship. Building explanatory or predictive models on such correlations therefore risks describing an artifact of time rather than a real association. Indeed, national Korean adolescent mental health indices have been shown to correlate very strongly with unrelated social indices such as the Gini coefficient over comparable periods [14], illustrating how readily spurious ecological co-movement arises.

This study uses 21 years (2005–2025) of Korean national aggregate KYRBS data, obtained through KOSIS, to explicitly separate (a) associations attributable to a shared secular trend from (b) associations that persist after the trend is statistically removed. We focus on the relationship between adolescent suicide attempts and ten commonly cited behavioral, psychological, and somatic-disease correlates, and—consistent with prior calls for multivariable modeling [13]—we additionally fit an exploratory path model to visualize the joint structure of the year-adjusted associations. All analyses and conclusions are strictly ecological (population-level); no individual-level or causal inference is intended.

## 2. Materials and Methods

### 2.1. Data Source and Study Design

This is a retrospective ecological (population-level) time-series analysis of publicly available, pre-aggregated annual national statistics. Data were obtained from the Korean Statistical Information Service (KOSIS; https://kosis.kr), which disseminates results of the KYRBS—an annual nationwide, self-administered, anonymous survey of middle- and high-school students conducted by the Korea Disease Control and Prevention Agency (KDCA) since 2005 [15]. The KYRBS uses a stratified, multistage, clustered sampling design representative of Korean middle- and high-school students nationwide; annual analytic samples ranged from approximately 51,850 to 75,643 students, with published participation rates consistently above 94% [15]. In more detail, the survey targets students aged approximately 12–18 years across middle-school grades 7–9 and high-school grades 10–12, drawn from roughly 400 middle and 400 high schools (schools as the primary sampling unit, one classroom per grade as the secondary unit); all students in a selected classroom are eligible to complete the anonymous, self-administered online questionnaire during regular class time [15]. Published national estimates incorporate the survey’s stratification, clustering, and post-stratification sampling weights and therefore reflect weighted, nationally representative prevalence rather than unweighted sample proportions [15]. For each indicator we extracted the national total (‘전체–전체–소계’) annual prevalence estimate (percentage) for all available years. No individual-level or identifiable data were accessed; only pre-aggregated summary statistics published by KOSIS were used.

### 2.2. Variables

The primary outcome was the annual national suicide attempt rate (percentage of students reporting a suicide attempt in the past 12 months, 2005–2025). Candidate correlates were perceived stress rate, depressive mood experience rate, self-reported obesity rate, body image distortion recognition rate, physician-diagnosed atopic dermatitis, allergic rhinitis and asthma, breakfast skipping rate, frequent fast food intake, and vigorous physical activity. KYRBS indicators are derived from standardized self-report items. Perceived stress represents the percentage of individuals answering that they feel stress ‘very much’ or ‘much’ in daily life; depressive mood represents the percentage of those reporting sadness or hopelessness severe enough to interrupt daily activities for ≥2 consecutive weeks in the past 12 months; and suicide attempt represents the percentage of those reporting ≥1 attempt in the past 12 months. Body image distortion is the percentage who perceive their body shape as ‘slightly or very fat’, and frequent fast food intake is the percentage consuming fast food ≥3 times per week. Atopic dermatitis, allergic rhinitis, and asthma are each ascertained from an affirmative response to ‘Have you ever been diagnosed with [condition] by a doctor?’ (lifetime physician diagnosis, not necessarily active in the survey year), and breakfast skipping is the percentage reporting no breakfast on ≥5 of the preceding 7 days. These are national prevalence indicators rather than validated psychometric scale scores, and item wording has been kept largely stable by the KDCA, although some items were revised or introduced in different years (see below), which we treat as a limitation (Section 4.4).

Year ranges reflect availability: perceived stress, depressive mood, suicide attempt and breakfast skipping (2005–2025); obesity (2006–2025); body image distortion (2008–2025); atopic dermatitis, allergic rhinitis and asthma (2007–2024); fast food intake (2009–2025); and vigorous physical activity (20 min/day, ≥3 days/week; 2005–2021, discontinued after a 2022 questionnaire revision). Years without an overlapping observation for a given pair of variables were excluded pairwise (available-case analysis).

### 2.3. Statistical Analysis

All analyses were performed in Python (v3.12; pandas, NumPy, SciPy, statsmodels, semopy). Throughout, |r| denotes the absolute value of the Pearson product-moment correlation coefficient. Because the number of annual observations is small (*n* = 17–21), we first assessed the distributional assumptions underlying parametric correlation: the calendar-year-detrended residuals of the suicide, stress, and depression series were approximately normally distributed (Shapiro–Wilk W = 0.94–0.95, all *p* > 0.26), supporting the use of Pearson correlations; nonetheless, we report Spearman rank-based correlations and permutation-based tests as distribution-free robustness checks (below).

First, raw Pearson correlation coefficients between the suicide attempt rate and each candidate correlate were computed using all pairwise-available years. Second, because most series exhibited a strong monotonic trend, each series was detrended by regressing it on calendar year and retaining the residuals; Pearson correlations between detrended residuals (year-adjusted partial correlations) were then computed. As robustness checks for these partial correlations, we additionally computed Spearman rank-based partial correlations and exact/Monte-Carlo permutation tests (10,000 permutations of the residualized indicator series). Third, ordinary least-squares multiple regression modeled the suicide attempt rate as a function of stress and depression, with and without an explicit year term; a hierarchical model then tested whether stress and depression added explanatory power beyond calendar year (ΔR^2^ with a nested F-test). We emphasize that these regression models use the observed (raw) suicide attempt series as the dependent variable and include calendar year as an explicit covariate; the year-detrended residual series (used for the partial correlations) is not used as the outcome here, which is why calendar year can and does contribute explanatory variance in the regression models. Because R^2^ is upward-biased in small samples, we report adjusted R^2^ alongside R^2^ and interpret magnitudes cautiously; we quantified collinearity between stress and depression using variance-inflation factors (VIF).

Fourth, as an exploratory step and following prior multivariable approaches [13], an observed-variable structural equation (path) model was fitted to the standardized, year-adjusted series to visualize the joint structure of associations among stress, depression, atopic dermatitis, obesity, and suicide attempts; standardized path coefficients are reported. Model identifiability was checked by counting sample moments and free parameters: with five observed variables, the covariance matrix provides 15 non-redundant moments, and the specified model estimates 15 free parameters (7 directed paths, 5 variances, and 3 exogenous covariances), so the model is exactly just-identified (df = 0, saturated). A just-identified model reproduces the observed covariances perfectly and therefore yields no testable global fit; combined with the small number of annual observations, this is why the path model is presented descriptively rather than as a confirmatory test. Because it contains no latent variables and is saturated, this observed-variable path model is statistically equivalent to a recursive system of ordinary-least-squares regressions—one per endogenous variable—so it carries no additional large-sample requirement beyond that of the individual regressions already reported in Section 3.2, and every path coefficient is estimated on *n* = 18 complete, pairwise-detrended annual observations. Finally, univariate damped-trend exponential-smoothing (Holt) models were fitted for suicide attempt, stress, and depression and extrapolated to 2026–2030 with approximate 95% intervals; this forecast is reported with explicit caveats (Section 4.4). Statistical significance was set at two-tailed *p* < 0.05.

## 3. Results

### 3.1. Raw Versus Year-Adjusted Correlations

Table 1 summarizes the raw and year-adjusted (partial) correlation of each candidate indicator with the national adolescent suicide attempt rate. In raw correlations, most indicators reached statistical significance, with |r| ranging up to 0.92 (depression). After removing the shared linear time trend, only perceived stress (partial r = 0.817, *p* < 0.001) and depressive mood (partial r = 0.813, *p* < 0.001) remained strongly and significantly associated with the suicide attempt rate. Atopic dermatitis (partial r = −0.640, *p* = 0.004) and asthma (partial r = −0.507, *p* = 0.032) remained significant but attenuated and in a direction that warrants cautious interpretation (Section 4.3), and breakfast skipping remained marginally significant. Associations for obesity, body image distortion, fast food intake, allergic rhinitis, and vigorous physical activity were no longer distinguishable from zero, indicating that their raw correlations were attributable to a shared secular trend rather than an independent relationship. The two robust psychological associations were confirmed by the distribution-free checks: Spearman rank-based partial correlations were ρ = 0.67 (stress, *p* = 0.001) and ρ = 0.78 (depression, *p* < 0.001), and permutation tests gave *p* = 0.0001 and *p* = 0.0002, respectively (Table 1).

**Figure 1:**
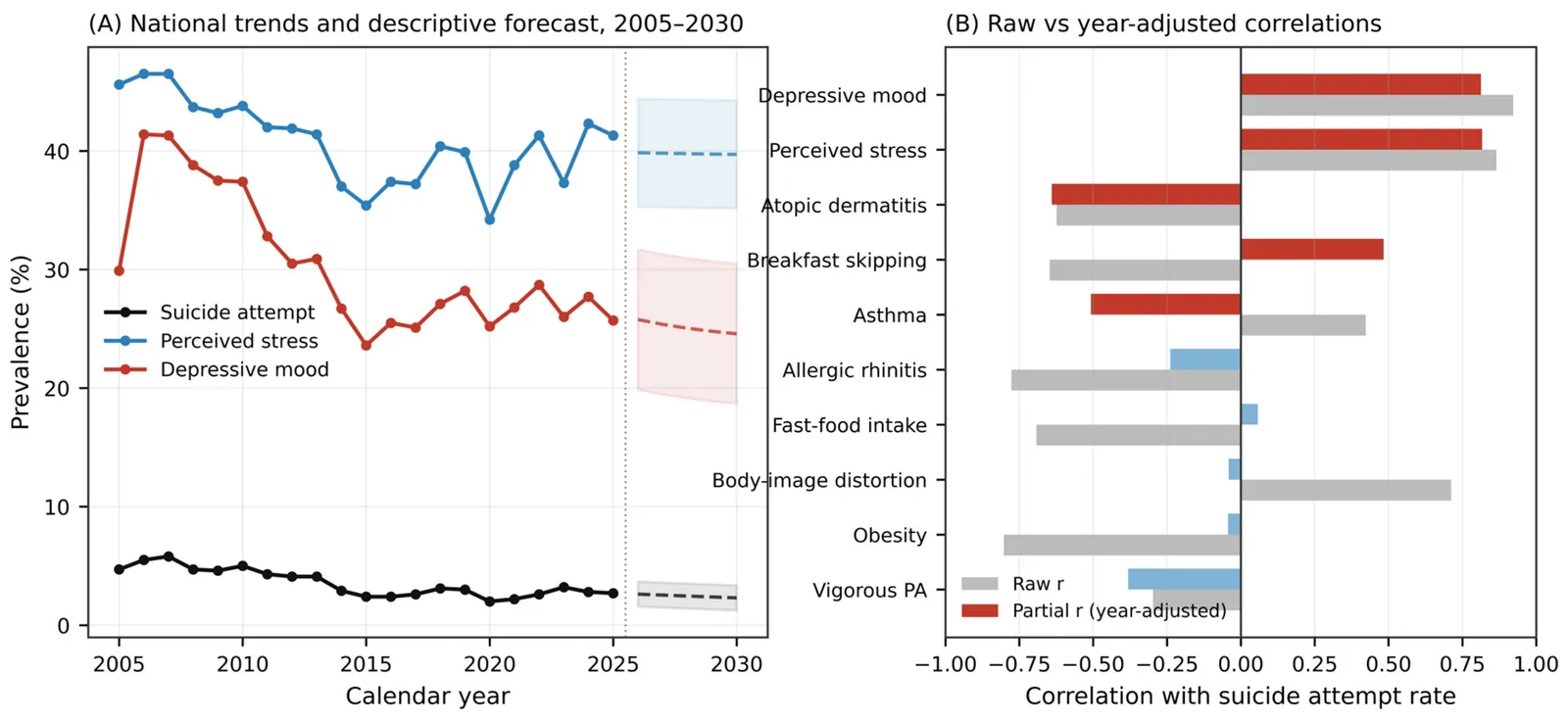
(**A**) National trends (2005–2025) in perceived stress, depressive mood, and suicide attempt rate among Korean adolescents, with a damped-trend exponential-smoothing forecast to 2026–2030 (dashed lines, shaded intervals). (**B**) Raw versus year-adjusted (partial) correlation of ten candidate indicators with the suicide attempt rate; gray bars denote raw correlations, and red and blue bars denote significant and non-significant year-adjusted (partial) correlations, respectively; the vertical dotted line in (**A**) separates observed values from the descriptive forecast. Data source: KOSIS/KDCA Korea Youth Risk Behavior Survey.

**Figure 2:**
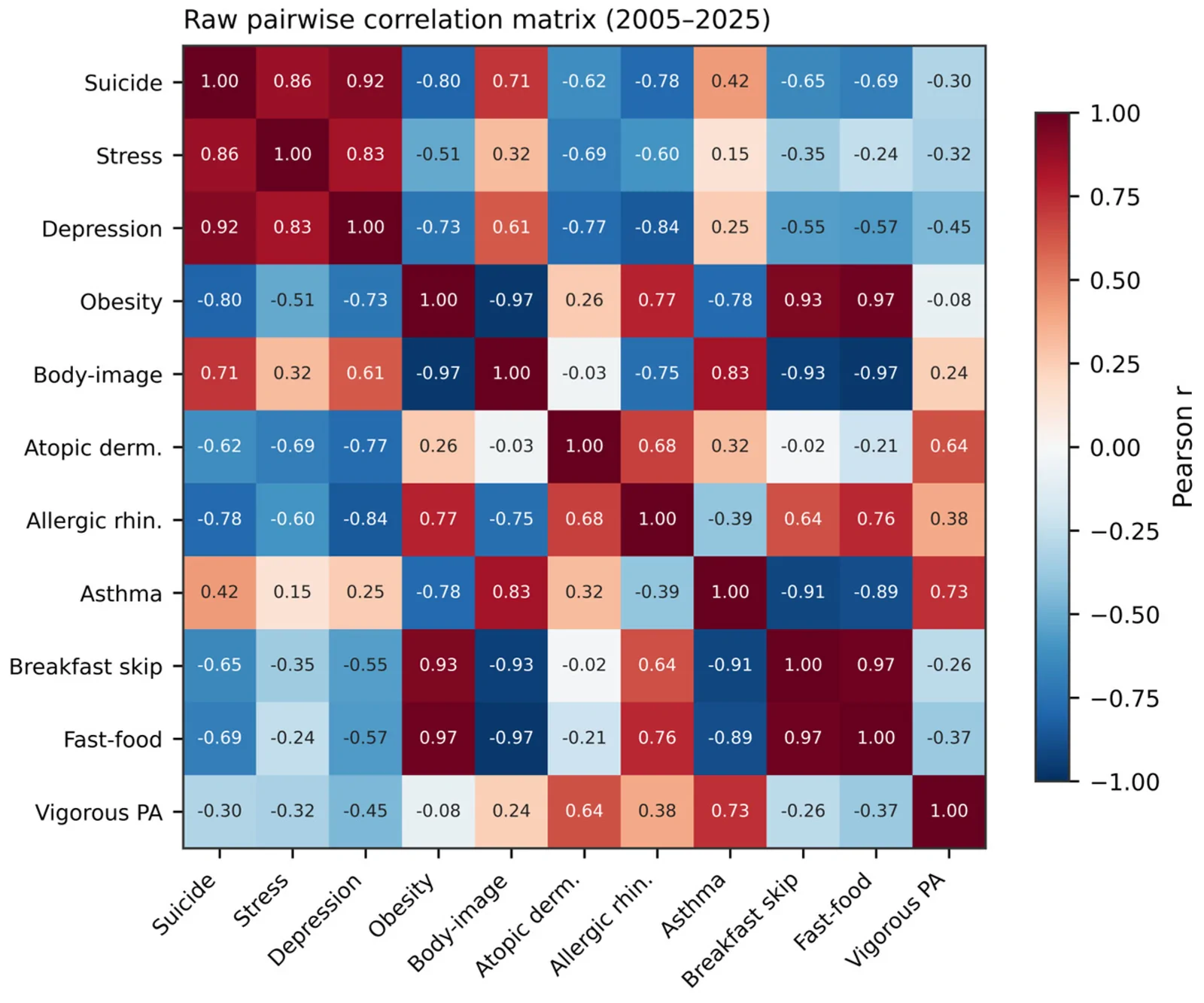
Raw pairwise Pearson correlation matrix among the eleven national adolescent health-behavior indicators examined, 2005–2025 (pairwise-available years).

**Figure 3:**
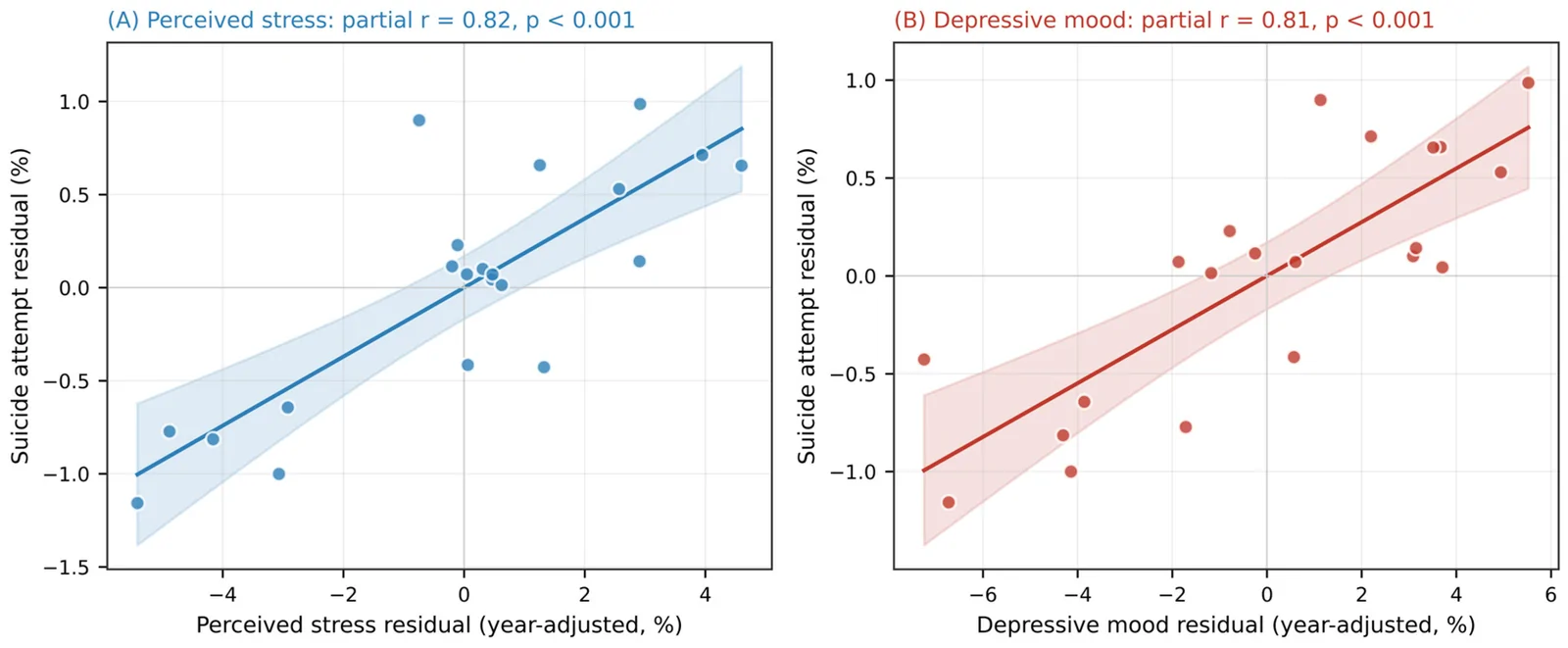
Year-adjusted (detrended) partial-regression scatter plots of the two robust psychological correlates against the national suicide attempt rate (*n* = 21 years). Each point represents one calendar year after both variables were residualized on calendar year; lines are OLS fits with 95% confidence bands. (**A**) Perceived stress (partial r = 0.817). (**B**) Depressive mood (partial r = 0.813).

Figure 1B contrasts the raw and year-adjusted coefficients for all ten indicators. The full pairwise correlation matrix among all indicators is shown in Figure 2. Figure 3 visualizes the two robust year-adjusted associations: the detrended residuals show clear positive linear relationships with the suicide attempt residual for both perceived stress (partial r = 0.817) and depressive mood (partial r = 0.813), confirming that these associations reflect genuine year-to-year co-variation rather than parallel long-term drift.

### 3.2. Multiple and Hierarchical Regression

Table 2 presents the regression of suicide attempts on stress and depression. In the unadjusted joint model, both stress (β = 0.106, *p* = 0.039) and depression (β = 0.135, *p* < 0.001) were independent significant predictors (R^2^ = 0.882, adjusted R^2^ = 0.869). Adding an explicit year term improved fit (R^2^ = 0.935, adjusted R^2^ = 0.923), and both stress (β = 0.110, *p* = 0.008) and depression (β = 0.079, *p* = 0.010) remained independently significant. In the hierarchical model, calendar year alone explained 70.6% of the variance, and adding stress and depression increased this to 93.5% (ΔR^2^ = 0.229; nested F(2,17) = 29.74, *p* < 0.001). The correlation between stress and depression was high (r = 0.829), but the associated variance-inflation factors were moderate (VIF = 3.2 for each), below conventional thresholds of concern (VIF > 5–10); both predictors therefore remained independently interpretable, although the precise magnitude of each coefficient should still be read with the shared variance in mind. We report adjusted R^2^ throughout because R^2^ is upward-biased with only 21 annual observations; the close agreement between R^2^ (0.935) and adjusted R^2^ (0.923) indicates limited overfitting, and much of the raw explanatory power reflects the strong shared secular trend (year alone R^2^ = 0.706) rather than the incremental psychological signal (ΔR^2^ = 0.229).

**Figure 4:**
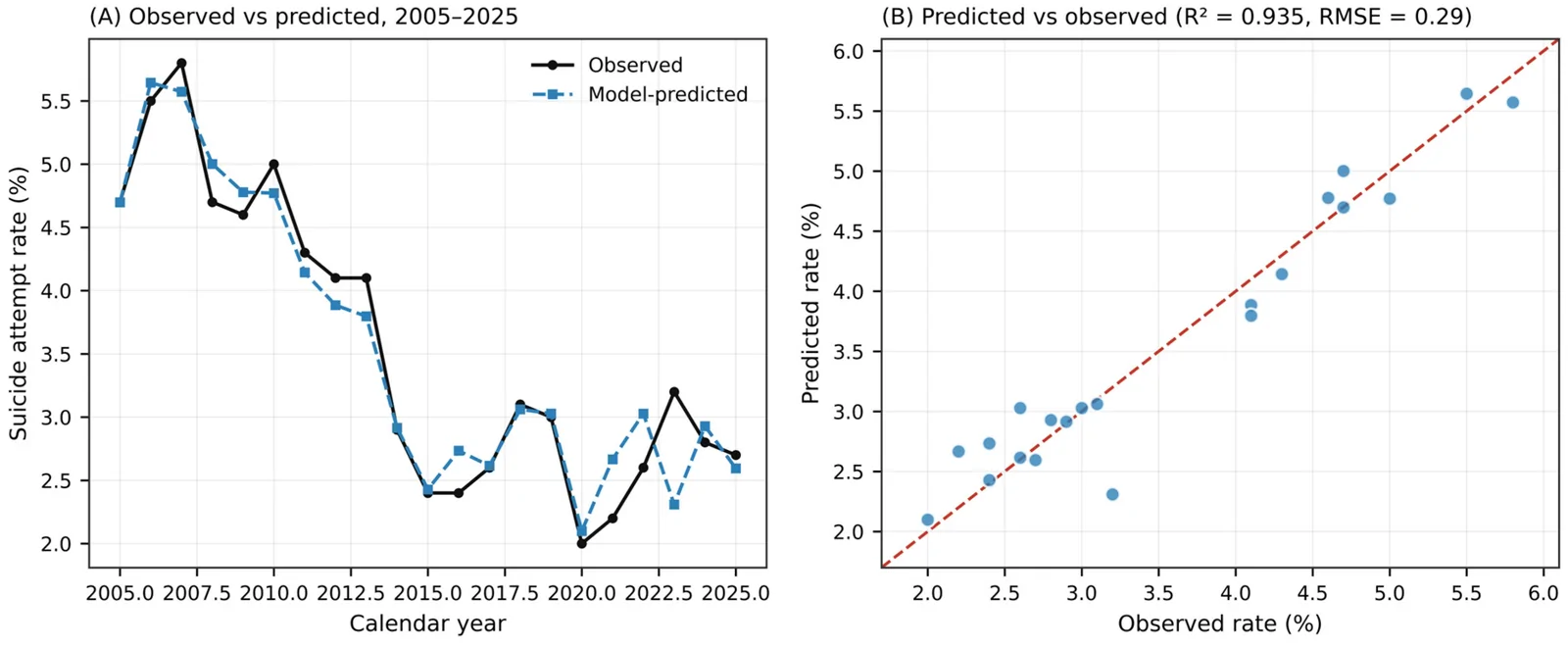
Fit of the year-adjusted multiple-regression model (suicide attempt ~ perceived stress + depressive mood + calendar year; *n* = 21). (**A**) Observed versus model—predicted rate, 2005–2025. (**B**) Predicted versus observed with the identity (y = x) line; R^2^ = 0.935.

The overall fit of the year-adjusted model is shown in Figure 4: predicted values closely tracked the observed series across all 21 years (R^2^ = 0.935; root-mean-square error = 0.29 percentage points), reproducing both the post-2012 decline and the 2020 pandemic-era trough, with no systematic bias.

### 3.3. Exploratory Path (SEM) Model

**Figure 5:**
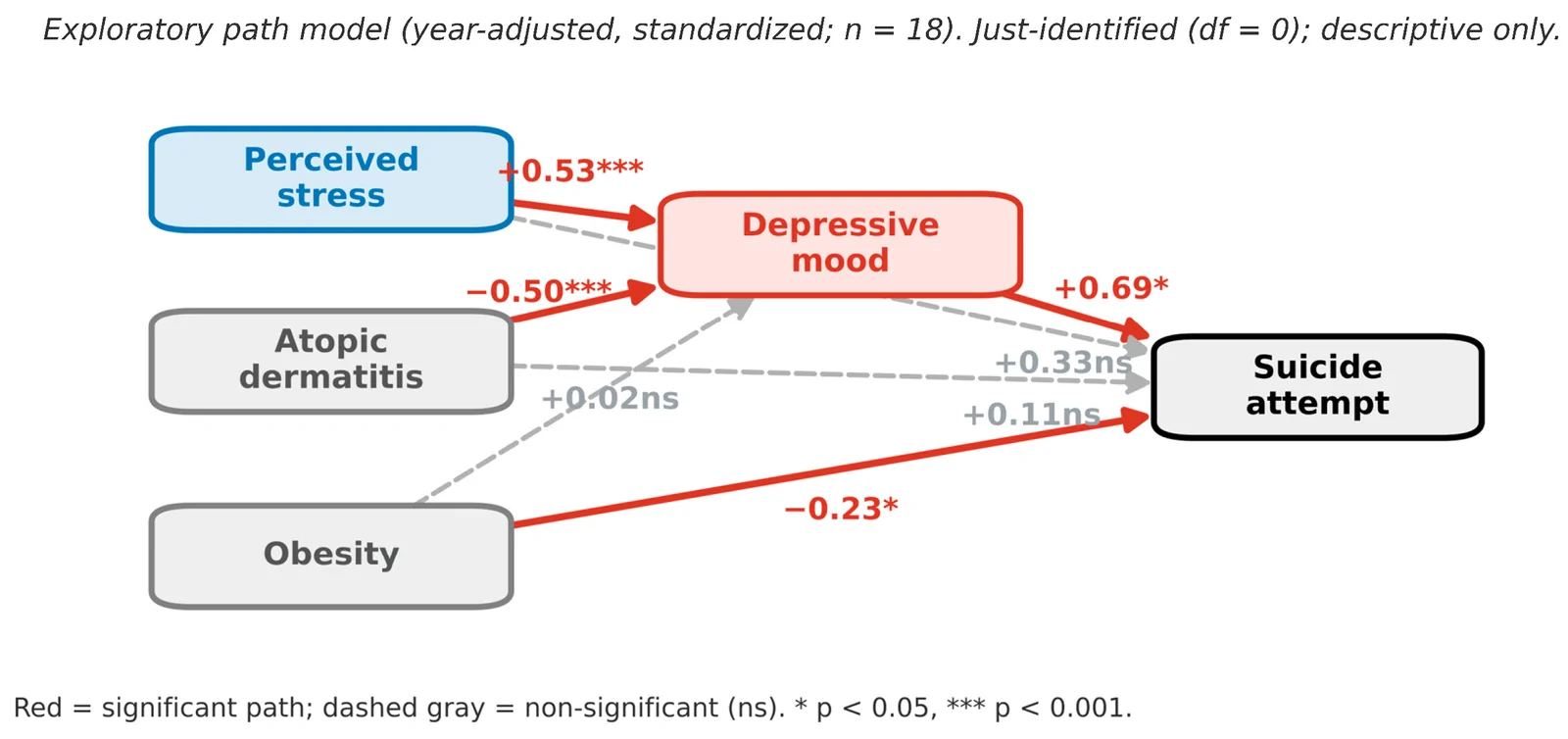
Exploratory path model of the year-adjusted, standardized associations among perceived stress, depressive mood, atopic dermatitis, obesity, and suicide attempts (*n* = 18). Values are standardized path coefficients; solid colored paths are significant (*p* < 0.05) and dashed gray paths are non-significant. Red paths denote significant associations, and paths marked ‘ns’ are non-significant. The model is just-identified (df = 0); global fit indices are not defined and are presented descriptively.

Table 3 and Figure 5 present the exploratory observed-variable path model fitted to the standardized, year-adjusted series (*n* = 18, 2007–2024). As noted in Section 2.3, this model is exactly just-identified (df = 0, saturated), so it reproduces the observed covariances without testable global fit and is interpreted descriptively. The psychological pathway was clearly the dominant structure: perceived stress strongly predicted depressive mood (standardized β = 0.529, *p* < 0.001), which in turn predicted the suicide attempt rate (β = 0.687, *p* = 0.022), while the direct stress → suicide path was not significant (β = 0.332, *p* = 0.114), consistent with depression partially mediating the stress–suicide relationship. In contrast, the somatic paths were either non-significant (obesity → depression, β = 0.023; atopic dermatitis → suicide, β = 0.106) or reached nominal significance in a direction opposite to established individual-level findings (atopic dermatitis → depression, β = −0.501; obesity → suicide, β = −0.228), which we interpret cautiously as small-sample and detrending artifacts (Section 4.3).

### 3.4. Descriptive Forecast, 2026–2030

A damped-trend exponential-smoothing forecast (Figure 1A) projected the national suicide attempt rate to remain broadly stable, at approximately 2.6% in 2026, declining gently to 2.3% by 2030; perceived stress was projected at approximately 40% and depressive mood at approximately 25–26% over the same period. These projections are descriptive only and should not be interpreted as validated forecasts (Section 4.4).

## 4. Discussion

This national time-series analysis illustrates a point that is important but easily overlooked in ecological studies of adolescent health. Over a 21-year window in which most indicators of Korean adolescent health and behavior have changed monotonically, almost any two series will appear significantly correlated regardless of whether a substantive relationship exists between them. Once the shared secular trend was removed, most raw associations with suicide attempts—including obesity, body image distortion, fast food intake, and allergic rhinitis—were no longer distinguishable from zero. Only perceived stress and depressive mood retained a strong, independent association, replicated across correlation, multiple and hierarchical regression, and the exploratory path model, and confirmed by rank-based and permutation tests.

### 4.1. Comparison with Prior Individual-Level Studies

The finding that perceived stress and depressive mood are the most robust correlates of adolescent suicide attempts is consistent with individual-level KYRBS research. Kim et al. found that stress arising from family and home was independently associated with suicidal ideation and attempts after adjustment for covariates [2], and comparison studies before and during the COVID-19 pandemic similarly identified stress and depressive symptoms as central to suicidality [12]. Woo et al. reported long-term national trends (2005–2021) in adolescent sadness and suicidality that declined before the pandemic and reversed thereafter [1], a trajectory closely matching the series observed here. The high raw ecological correlation between mental health outcomes and unrelated social indices reported by Kim et al. [14] reinforces the need for the year-adjustment applied here before attributing substantive meaning to any raw ecological correlation.

### 4.2. Somatic and Behavioral Factors

At the individual level, atopic dermatitis and other allergic diseases are associated with higher—not lower—rates of depression and suicidality among adolescents [6,7,9], and obesity and adverse weight perception are likewise associated with greater depressive symptoms [10]. The present ecological analysis does not contradict, and cannot test, those individual-level findings. Rather, it shows that at the population level the annual national prevalence of these somatic conditions does not track the annual suicide attempt rate once the shared secular trend is removed. This is an expected consequence of the ecological design: national prevalence of atopic dermatitis or obesity is driven by many determinants (diagnostic practice, environmental exposure, body-composition trends) that need not move in step with population suicide risk, even when an individual-level association exists. Crucially, the absence of a population-level (ecological) association does not imply that these somatic conditions are unrelated to suicide risk in individual adolescents; inferring the latter from the former would itself be an ecological fallacy. The divergence between robust individual-level positive associations and the null or artifactually negative year-adjusted ecological associations observed here is itself an instructive illustration of that fallacy.

### 4.3. Interpretation of the Path Model and Attenuated Associations

The exploratory path model reproduced the psychological pathway (stress → depression → suicide) as the only theoretically coherent and individually corroborated structure. The nominally significant but reversed-direction paths for atopic dermatitis → depression and obesity → suicide should not be interpreted as protective effects: they contradict well-established individual-level evidence [6,7,10]. With only 18 detrended annual observations and several indicators tested, some spurious partial associations are expected, and detrending residuals from short, noisy series can introduce artifactual short-run co-movement. Because the model is just-identified (df = 0), it cannot be evaluated by global fit and offers no test of causal structure. We therefore regard the stress and depression associations (partial r ≈ 0.81–0.82, *p* < 0.001, replicated across four analytic approaches and two distribution-free checks) as the only findings that meet a reasonable threshold of robustness, and we present the path model strictly as a descriptive visualization.

### 4.4. Limitations

Several limitations apply. First, all analyses use pre-aggregated national annual prevalence estimates rather than individual-level data; associations observed at the ecological level cannot be assumed to hold at the individual level, and causal interpretation is precluded. Second, the sample size for time-series inference is small (*n* = 17–21 annual observations), which limits power, restricts the number of covariates that can be estimated simultaneously, and makes a latent-variable SEM inappropriate; the path model is consequently exploratory and just-identified. We mitigated, but cannot eliminate, small-sample concerns by reporting adjusted R^2^, Spearman rank-based correlations, and permutation tests alongside the parametric results. Third, several KYRBS items underwent question-wording or methodology changes (e.g., the physical-activity item was revised in 2022 and could not be extended past 2021 comparably), potentially introducing discontinuities unrelated to genuine behavioral change. Fourth, the 2026–2030 forecast is descriptive only and should not be read as a validated forecast. Finally, as with all KYRBS research, outcomes are self-reported and subject to reporting and recall bias, particularly for a sensitive outcome such as suicide attempts.

## 5. Conclusions

In this 21-year national time-series analysis of Korean adolescents, perceived stress and depressive mood showed a robust population-level association with suicide attempts that persisted after accounting for the shared secular trend affecting nearly all adolescent health indicators, whereas associations for obesity, body-image distortion, diet, physical activity, and most allergic/atopic conditions did not. These findings support continued prioritization of psychological stress and depressive symptoms as targets for adolescent suicide-prevention policy in South Korea. They should not, however, be over-generalized: because the analysis is ecological, the absence of a population-level association for somatic and behavioral factors does not establish that these factors are unimportant for suicide risk in individual adolescents, and the results should not be read as diminishing the well-documented individual-level links between somatic conditions and adolescent mental health. Prospective, individual-level longitudinal studies remain necessary to establish the direction and mechanism of the observed associations.

## Figures and Tables

**Table 1:** Raw versus year-adjusted (partial) correlation of each indicator with the national adolescent suicide attempt rate. Spearman rank-based partial correlations and permutation *p*-values added as distribution-free robustness checks.

Indicator	*n*	Raw r	Partial r (Year-Adjusted)	*p* (Partial)	Spearman Partial ρ	Permutation *p*
Perceived stress	21	0.865 ***	0.817 ***	<0.001	0.673	0.0001
Depressive mood	21	0.922 ***	0.813 ***	<0.001	0.784	0.0002
Atopic dermatitis	18	−0.623 **	−0.640 **	0.004	−0.595	0.0053
Breakfast skipping	21	−0.647 **	0.484 *	0.026	0.492	0.0286
Asthma	18	0.423 ns	−0.507 *	0.032	−0.406	0.0304
Obesity (self-report)	20	−0.802 ***	−0.043 ns	0.856	0.002	0.860
Body image distortion	18	0.712 ***	−0.041 ns	0.872	0.098	0.866
Fast food intake (≥3×/wk)	17	−0.692 **	0.058 ns	0.826	−0.034	0.822
Allergic rhinitis	18	−0.777 ***	−0.239 ns	0.340	−0.181	0.339
Vigorous PA (20 min, ≥3 d/wk)	17	−0.297 ns	−0.381 ns	0.131	−0.407	0.127

*** *p* < 0.001; ** *p* < 0.01; * *p* < 0.05; ns = not significant. |*r*| denotes the absolute Pearson coefficient. Raw and partial correlations use all pairwise-available years. Spearman partial ρ and permutation *p* (10,000 permutations) computed on the year-adjusted residuals.

**Table 2:** Multiple and hierarchical regression of the national suicide attempt rate on perceived stress and depressive mood (*n* = 21 years).

Term	β	SE	*p*
Model 1 (R^2^ = 0.882; adj. R^2^ = 0.869)			
Perceived stress	0.106	0.048	0.039
Depressive mood	0.135	0.030	<0.001
Model 2, year-adjusted (R^2^ = 0.935; adj. R^2^ = 0.923)			
Calendar year	−0.065	0.018	0.002
Perceived stress	0.110	0.037	0.008
Depressive mood	0.079	0.027	0.010
Hierarchical model (ΔR^2^ test)			
Step 1: year only	R^2^ = 0.706	—	—
Step 2: + stress + depression	R^2^ = 0.935	ΔR^2^ = 0.229	<0.001

β, unstandardized regression coefficient; SE, standard error; ΔR^2^, change in R^2^ relative to the year-only model. Model 2 uses the observed (raw) suicide series with calendar year as a covariate; VIF for stress and depression = 3.2 (each).

**Table 3:** Standardized path coefficients from the exploratory path model on the year-adjusted, standardized series (*n* = 18).

Path	Std. β	SE	*p*
Perceived stress → Depressive mood	0.529	0.107	<0.001
Atopic dermatitis → Depressive mood	−0.501	0.110	<0.001
Obesity → Depressive mood	0.023	0.084	0.788
Depressive mood → Suicide attempt	0.687	0.301	0.022
Perceived stress → Suicide attempt	0.332	0.210	0.114
Atopic dermatitis → Suicide attempt	0.106	0.206	0.608
Obesity → Suicide attempt	−0.228	0.108	0.035

Std. β, standardized path coefficient. Model estimated on detrended, standardized series; just-identified (df = 0, saturated), so global fit indices are not defined (see Section 2.3 and Section 4.3).

## Data Availability

The data analyzed in this study are publicly available as aggregate annual national statistics via the Korean Statistical Information Service (KOSIS; https://kosis.kr), reflecting results of the Korea Youth Risk Behavior Survey conducted by the Korea Disease Control and Prevention Agency. Individual-level data were not accessed. The merged annual dataset compiled for this analysis is available from the corresponding author upon reasonable request.
